# Association of circulating CTRP9 with soluble adhesion molecules and inflammatory markers in patients with type 2 diabetes mellitus and coronary artery disease

**DOI:** 10.1371/journal.pone.0192159

**Published:** 2018-01-30

**Authors:** Nariman Moradi, Reza Fadaei, Solaleh Emamgholipour, Elham Kazemian, Ghodratollah Panahi, Siamak Vahedi, Lotfolah Saed, Soudabeh Fallah

**Affiliations:** 1 Department of Clinical Biochemistry, Faculty of Medicine, Iran University of Medical Sciences, Tehran, Iran; 2 Department of Clinical Biochemistry, Faculty of Medicine, Kermanshah University of Medical Sciences, Kermanshah, Iran; 3 Department of Clinical Biochemistry, Faculty of Medicine, Tehran University of Medical Sciences, Tehran, Iran; 4 Department of Basic Sciences and Cellular and Molecular Nutrition, Faculty of Nutrition Sciences, Shahid Beheshti University of Medical Sciences, Tehran, Iran; 5 Department of Cardiology, Faculty of medicine. Kurdistan University of Medical Science, Sanandaj, Iran; 6 Department of Internal Medicine, Faculty of Medicine, Kurdistan University of Medical Sciences, Sanandaj, Iran; 7 Research center of Pediatric Infectious Disease, Rasool Akram Hospital, Iran University of Medical Sciences, Tehran, Iran; East Tennessee State University, UNITED STATES

## Abstract

C1q/TNF-related protein 9 (CTRP9) is a paralogue of adiponectin with known favorable effects on lipid and glucose metabolism. A potential role of CTRP9 for regulation of endothelium function has been suggested by previous studies. However, no studies have examined the relation between serum CTRP9 levels and adhesion molecules in patients with type 2 diabetes mellitus (T2DM) and coronary artery disease (CAD). The present study was conducted on 337 subjects who underwent coronary angiography and were categorized into four groups according to the presence of CAD and T2DM (control, CAD, T2DM and CAD+T2DM). Serum levels of CTRP9, adiponectin, sICAM-1, sVCAM-1, sE-Selectin, IL-6 and TNF-α were measured. It was found that the circulating CTRP9 levels were independently associated with increased risk of CAD and T2DM in addition to elevated levels of serum CTRP9 in CAD, T2DM and CAD+T2DM groups. A significant association of serum CTRP9 levels with adhesion molecules in CAD and T2DM patients as well as serum TNF-α levels in CAD individuals was noted. A significant relation between the circulating levels of CTRP9 and HOMA-IR in T2DM subjects was also observed. The results revealed increased circulating levels of CTRP9 in T2DM and CAD individuals which suggests a compensatory response to insulin resistance, inflammatory milieu and endothelial dysfunction; however, more studies are needed to confirm this.

## Introduction

Adipose tissue is a highly active endocrine organ that is responsible for the synthesis and secretion of several hormones, such as bioactive molecules known as adipokines [1]. In recent years, considerable research has been devoted to understanding the biology of adipokines and their potential role in obesity-related diseases such as diabetes and cardiovascular disease (CVD) [2,3]. Adipokines have been identified as having diverse functional roles in lipid and glucose metabolism and in inflammation, along with the pathogenic processes of many diseases [4,5].

Adiponectin, a well-known adipokine, exerts a positive role in regulating glucose and lipid metabolism. It has been suggested that the dysregulation of adiponectin production contributes to the development of CVD and type 2 diabetes mellitus (T2DM) [4]. The C1q TNF related protein (CTRP) family is a newly discovered paralogue of adiponectin [6]. This family has 15 members (CTRP1 to CTRP15) with related structures and diverse functions [7]. Among them, CTRP9 has the highest amino acid identity to adiponectin and is secreted as a glycoprotein from adipose tissue [8]. It has been shown that overexpression of CTRP9 decreases fasting insulin and glucose levels in mice [9]. Conversely, deletion of CTRP9 decreases insulin sensitivity and increases food intake [10]. Moreover, TNF-α inhibits CTRP9 expression in H9c2 cells [11].

Several beneficial effects of CTRP9 for the cardiovascular system have been reported. It has higher vasoactive potency than adiponectin [12], has a protective role in remodeling after acute myocardial infarction [13], decreases inflammation [14] and inhibits vascular smooth muscle cell proliferation [15]. The association of plasma CTRP9 levels with atherosclerosis has been suggested by several lines of evidence [16–18]. For instance, Chang Hee Jung et al. reported independent association between CTRP9 and arterial stiffness [19]. Jing Wang et al. showed decreased serum levels of CTRP9 in patients with coronary atherosclerosis [16].

Conflicting results have been reported in relation to CTRP9 levels and metabolic factors. Some studies have found a positive association between CTRP9 and unfavorable metabolic factors such as body mass index (BMI) and insulin resistance [19,20], while the others described inverse associations [21]. Although the effect of CTRP9 on endothelium function has been pointed out by previous studies [10,22], there is no study on the association between CTRP9 and soluble adhesion molecules as markers of endothelial function in patients with T2DM and coronary artery disease (CAD). This study aimed to evaluate the association of circulating CTRP9 levels with CAD and T2DM as well as the association between serum CTRP9 levels and soluble adhesion molecules as a marker of endothelium dysfunction in CAD and T2DM patients.

## Study population, materials and methods

### Study population

Participants aged 45–75 years were recruited from Rasoul-e-Akram Hospital in Tehran, Iran. This study was approved by the Ethics Committee of Iran University of Medical Sciences and was conducted in accordance with the Helsinki Declaration. Written consent was obtained from all study subjects. Patients who had at least one coronary vessel with >50% luminal stenosis were categorized as CAD patients as determined by a cardiologist. Subjects with <30% stenosis in angiography imaging were categorized as non-CAD.

American Diabetes Association (ADA) criteria were used for the diagnosis of T2DM [23]. Subjects with acute coronary syndrome and cardiovascular disease (peripheral artery, coronary artery and cerebrovascular disease) were excluded from the non-CAD group. Moreover, individuals with a history and evidence of stroke, myocardial infraction, kidney disease, cancer, autoimmune disease, chronic inflammation and those taking thiazolidinedione [11] were excluded from the study. Subjects who had smoked cigarettes in the last three months were considered to be smokers. Study participants were categorized into the following four groups according to the presence of CAD and T2DM: control (non-CAD and non-T2DM), CAD (CAD and non-T2DM), T2DM (T2DM and non-CAD) and CAD+T2DM (CAD and T2DM).

### Anthropometric measurement and laboratory assessment

The body mass index (BMI) was calculated by dividing the weight (kg) by height squared (m). The systolic blood pressure (SBP) and diastolic blood pressure (DBP) were measured in seated patients after 5 min of rest using a standard sphygmomanometer. After the subject had fasted overnight, blood samples were collected and the fasting blood glucose (FBG), total cholesterol (TC), high-density lipoprotein cholesterol (HDL-C), low-density lipoprotein cholesterol (LDL-C), triglycerides (TG), alanine amino transferase (ALT), aspartate amino transferase (AST) and creatinine (Cr) were measured using commercially available kits (Pars Azmoon; Iran). Serum insulin levels were assessed by ELISA kit (Monobind; USA). Homeostasis model assessment for insulin resistance (HOMA-IR) was calculated using the formula (FBG [mg/dL]) × (fasting blood insulin [μU/ml])/405).

#### Measurement of circulating adipokines, cytokines and adhesion molecules

Serum levels of TNF-α (Cat #DTA00C), IL-6 (Cat #HS600B), ICAM-1 (Cat #DCD540), VCAM-1 (Cat #DVC00) and E-Selectin (Cat #DSLE00) were measured using ELISA kits (Quantikine; R&D Systems; USA). Minimum detectable doses of TNF-α and IL-6 were 1.6 and 0.7 pg/ml, respectively. The intra- and inter-assay coefficients of variation (CV) of ICAM-1, VCAM-1 and E-Selectin were <7%, <6.5% and <7.5%, respectively. Circulating adiponectin levels were measured by an ELISA kit (Adipogen; South Korea; Cat #AG-45A-0001YEK-KI01) with intra- and inter-assay variations of 4.6% and 4.4%, respectively. Serum levels of CTRP9 were measured using an ELISA kit (USCN Life Science; USA; Cat #SER877Hu) (Intra-assay: CV = 3.4%; Inter-assay: CV = 4.3%).

#### Statistical analysis

Categorical variables were presented as frequencies and percentages and compared using the chi-square test. Shapiro-Wilkes testing was conducted to determine the normal distribution of quantitative variables. Variables with normal distribution were reported as mean ± standard error of mean (SEM) and compared using student's t-test or one-way ANOVA followed by Bonferroni post hoc. Non-normally distributed variables were shown as median ± inter quartile range (IQR) and tested using Mann–Whitney U or Kruskal-Wallis tests with the Bonferroni correction. Analysis of covariance (ANCOVA) was performed to control potential covariates. Logarithmic transformation carried out for non-normally distributed data and correlations between continuous variables were determined by Pearson’s correlation test. Multinomial logistic regression was conducted to evaluate the association between serum CTRP9 levels and disease conditions. Multiple linear regression analysis was run to assess the relations between CTRP9 levels and correlated variables.

## Results

Anthropometric and clinical characteristics of the study participants are presented in Table 1. No significant differences in age, sex or BMI were observed between groups. The number of smokers and individuals taking statins and antihypertensive medications were higher in the case groups than in the control. Anti-hyperglycemic medications were used only by T2DM patients (T2DM and CAD+T2DM groups). Details of medication use are given in S1 Table. Higher SBP, DBP and FBS values were found in the CAD+T2DM group compared to the control. The highest level of FBS and the lowest levels of insulin and the HOMA-IR index were observed in the control group. In addition, compared to T2DM and CAD+T2DM groups, CAD individuals had lower HOMA-IR indexes. Furthermore, the results of post hoc analysis showed a significant difference in circulating levels of TG, TC, LDL-C, HDL-C, ALT and AST between the case and control groups.

**Table 1:** Clinical and biochemical characteristics of study population.

Variables	Control(n = 80)	CAD (n = 157)	T2DM (n = 37)	CAD+T2DM (n = 63)	p value
Sex [male (%)]	58 (72.5)	112 (71.3)	21 (56.8)	45 (71.4)	0.318
Age (year)	57.03 ± 0.97	58.18 ± 0.62	58.51 ± 1.23	58.49 ± 1.13	0.661
BMI (kg/m2)N	25.8 ± 3.4	26.6 ± 3.9	26.7 ± 4.2	26.4 ± 4.1	0.435
Smoker [n (%)]	17 (21.2)	66 (42)	11 (29.7)	31 (49.2)	<0.001
SBP (mm Hg)	128.5 ± 16.2	132.4 ± 18.3	131.8 ± 18.8	138.4 ± 19.1^c^^**^	0.014
DBP (mm Hg)	79.5 ± 11.4	82.6 ± 13.0	81.2 ± 14.0	86.5 ± 13.6 ^c^^**^	0.015
FBG (mg/dl)	93.8 ± 11.7	95.2 ± 11.5	167.9 ± 23.7^b^^**^^,^^d^^**^	156.0 ± 22.4^c^^**^^,^^e^^**^^,^^f^^**^	<0.001
Insulin (μU/ml)	3.2 (2.1–5.5)	5.5 (2.8–8.6)^a^^**^	10.9 (8.9–12.8) ^b^^**^^,^^d^^**^	9.4 (7.0–12.2) ^c^^**^^,^^e^^**^	<0.001
HOMA-IR	0.78 (0.46–1.24)	1.35 (0.64–2.17)^a^^**^	4.15 (3.45–5.44)^b^^**^^,^^d^^**^	3.53 (2.51–4.94)^c^^**^^,^^e^^**^	<0.001
Triglyceride (mg/dl)	121.8 ± 47.4	140.9 ± 48.9^a^^*^	148.9 ± 38.9^b^^*^	169.8 ± 63.6^c^^**^^,^^e^^**^	<0.001
Total Cholesterol (mg/dl)	170.4 ± 37.7	182.6 ± 46.0	181.4 ± 44.1	196.1 ± 45.7^c^^**^	0.008
LDL-C (mg/dl)	102 ± 30.6	110.3 ± 33.5	108.5 ± 37.2	124.0 ±35.2^c^^**^^,^^e^^*^	0.002
HDL-C (mg/dl)	45.9 ± 7.2	44.0 ± 10.1	42.2 ± 4.3	41.4 ± 6.0^c^^**^	0.009
Creatinine (mg/dl)	1.13 ± .18	1.15 ± .18	1.20 ± .15	1.19 ± .15	0.116
AST (U/l)	18.2 ± 5.5	21.5 ± 6.7^a^^*^	19.8 ± 5.4	19.7 ± 6.6	0.002
ALT(U/l)	18.2 ± 7.6	22.9 ± 8.4^a^^*^	18.3 ± 7.4	21.8 ± 8.3	<0.001
Antihypertensive medication [n (%)]	11 (13.8)	56 (35.7)	5 (13.5)	33 (52.4)	<0.001
Statin use [n (%)]	23 (28.7)	88 (56.1)	13 (35.1)	34 (54)	<0.001
Oral hypoglycemic agent [n (%)]	0	0	20 (54.1)	42 (66.7)	<0.001
Insulin ± Oral hypoglycemic agent [n (%)]	-	-	11 (29.7)	17 (27)	<0.001

a: Control and CAD groups were compared.

b: Control and T2DM groups were compared.

c: Control and T2DM+CAD groups were compared.

d: CAD and T2DM groups were compared.

e: CAD and T2DM+CAD groups were compared.

f: T2DM and CAD+T2DM groups were compared.

* P < 0.05

** P < 0.01.

### Serum concentrations of CTRP9, cytokines and adhesion molecules

**Fig 1:**
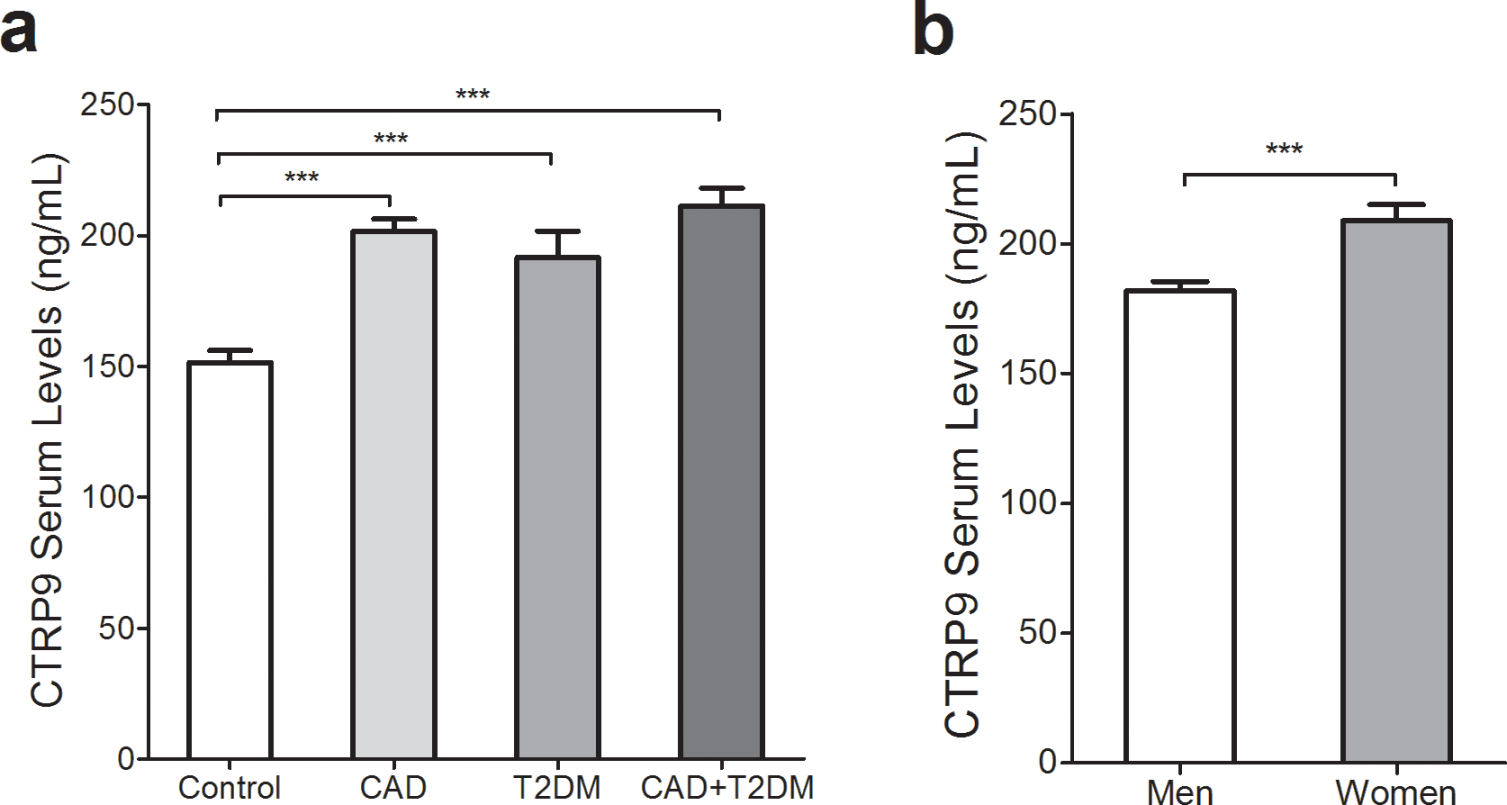
Serum levels of CTRP9 in control, CAD, T2DM and CAD+T2DM category. a) Serum CTRP9 levels were lower in controls (148.7 ± 4.0) than CAD (202.0 ± 4.9), T2DM (191.4 ± 10.1) and CAD+T2DM (211.2 ± 6.8). (all, p<0.001). b) Serum concentration of CTRP9 was higher in women (208.8 ± 6.2) compared to men (181.8 ± 3.7) (p<0.001).

Circulating CTRP9 levels were significantly higher in CAD (202.03 ± 4.89), T2DM (191.38 ± 10.13) and CAD+T2DM (211.19 ± 6.81) patients (p < 0.001) compared to control individuals (148.7 ±4.0) (Fig 1A). The results remained significant even after adjusting for age, BMI, sex, medication and adiponectin (S2 Table). Likewise, the results of multiple logistic regression indicate that serum CTRP9 concentration was independently associated with the increased risk of CAD (OR [CI] = 1.018 [1.009–1.026]; p < 0.001), T2DM (OR [CI] = 1.015 [1.005–1.025]; p = 0.003) and CAD+T2DM (OR [CI] = 1.021 [1.012–1.031]; p < 0.001) after adjustment for age, sex, BMI, adiponectin, IL-6 and TNF-α (Table 2). Serum levels of CTRP9 were significantly higher in women (208.7 ± 6.3) than men (181.8 ± 3.7; p < 0.001; Fig 1B).

**Table 2:** Odds ratios for development of CAD, T2DM and CAD+T2DM based on serumCTRP9 levels.

	CTRP9 serum levels	
	OR (95% Cl)	P value
**CAD group**		
Unadjusted model	1.022 (1.015–1.029)	<0.001
Model 1	1.018 (1.009–1.026)	<0.001
**T2DM group**		
Unadjusted model	1.018 (1.010–1.027)	<0.001
Model 1	1.015 (1.005–1.025)	0.003
**CAD+T2DM group**		
Unadjusted model	1.025 (1.017–1.032)	<0.001
Model 1	1.021 (1.012–1.031)	<0.001

Model 1: Adjusted for age, sex, BMI, adiponectin, IL-6 and TNF-α

**Fig 2:**
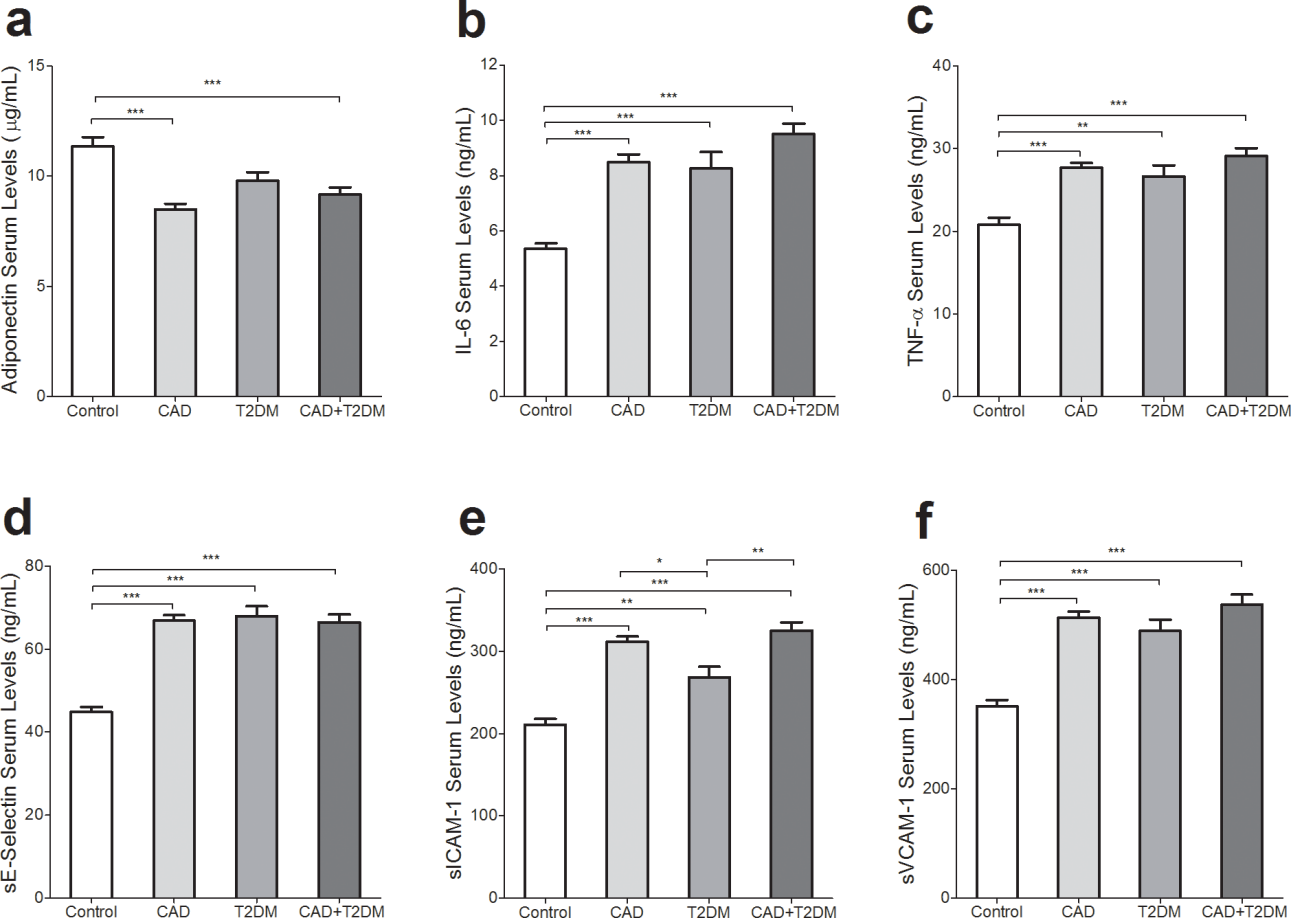
Circulating levels of adiponectin, inflammatory cytokines and soluble adhesion molecules in control, CAD, T2DM and CAD+T2DM category. a) A higher levels of serum adiponectin was demonstrated in controls compared to persons with CAD and CAD+T2DM (both, p<0.001). b) Serum concentration of IL-6 was higher in patients groups compared to controls (all, p<0.001). c) TNF-α concentration were higher in CAD, T2DM and CAD+T2DM category compared to control (all, p<0.001). d) Those with CAD, T2DM, and CAD+T2DM showed a higher serum levels of E-selectin compared to control group (all, p<0.001). e) sICAM-1 was higher in case groups compared to control (all, p<0.01). Also, higher serum levels of sICAM-1 were shown in CAD (p<0.05) and CAD+T2DM patients (p<0.01) compared to T2DM group. f) Serum level of sVCAM-1 had higher levels in patients compared to healthy individuals (all, p<0.001).

Circulating levels of adiponectin were lower in CAD (8.51 ± 0.24) and CAD+T2DM (9.17 ± 0.33) than in the control category (11.36 ± 0.42; p < 0.001; Fig 2A). Serum IL-6 was higher in concentration in CAD (8.5 ± 0.3), T2DM (8.3 ± 0.6) and CAD+T2DM (9.5 ± 0.4) than in the controls (5.3 ± 0.2; p < 0.001; Fig 2B). Circulating levels of TNF-α were lower in control subjects (20.82 ± 0.87) than in those with CAD (27.74 ± 0.54), T2DM (26.65 ± 01.32) and CAD+T2DM (29.12 ± 0.94) (p < 0.001, p < 0.01 and p < 0.001, respectively; Fig 2C). Serum sE-Selectin levels were also higher in CAD (66.9 ± 1.3), T2DM (68.0 ± 2.3) and CAD+T2DM (66.5 ± 1.9) patients compared to normal participants (44.9 ± 1.2; p < 0.001; Fig 2D). Compared with the CAD (311.5 ± 6.4), T2DM (268.0 ± 13.1) and CAD+T2DM (324.9 ±9.9) groups, a lower concentration of serum sICAM-1 was observed in the controls (210.7 ± 6.9; p < 0.001). Serum sICAM-1 was higher in the CAD+T2DM and CAD groups than in subjects with T2DM (p < 0.01 and p < 0.05, respectively; Fig 2E). Compared to normal subjects (352.5 ± 11.2), the CAD (513.5 ± 10.4), T2DM (489.2 ± 20.6) and CAD+T2DM (537.3 ± 18.0) subjects recorded higher concentrations of sVCAM-1 (p < 0.001; Fig 2F).

### Association of CTRP9 with biochemical and metabolic parameters

The results of the Pearson correlation of CTRP9 with anthropometric and metabolic parameters are shown in Table 3. Significant correlations were found between serum CTRP9 levels, BMI and glucose metabolism parameters (FBG, insulin and HOMA-IR) in the control group. The results of multiple stepwise linear regression showed that BMI (β [SE] = 2.9 [1.1]; p = 0.001) and HOMA-IR (β [SE] = 40.7[13.8]; p = 0.004) were independently associated with serum CTRP9 levels in normal subjects. In the CAD group, CTRP9 was significantly and positively correlated with inflammatory cytokines (IL-6 and TNF-α), adhesion molecules (sE-selectin, sICAM-1 and sVCAM-1) and was negatively associated with adiponectin (p < 0.01). The results of multiple stepwise linear regression analysis revealed that TNF-α (β [SE] = 83.8 [38.7]; p = 0.32), sE-Selectin (β [SE] = 0.794 [0.264]; p = 0.003), sICAM-1 (β [SE] = 0.138 [0.058]; p = 0.018), sVCAM-1 (β [SE] = 0.106 [0.035]; p = 0.003) and adiponectin (β [SE] = -4.822 [1.38]; p = 0.001) were independently associated with serum CTRP9 levels. In the T2DM group, CTRP9 showed significant positive correlations with BMI, FBG, insulin, HOMA-IR, sE-Selectin, sVCAM-1 and sICAM-1 (p < 0.05) and a negative association with adiponectin (p < 0.05); however; the correlations between CTRP9 and BMI and insulin and sICAM-1 disappeared after adjustment for adiponectin in the T2DM group. A multiple linear regression model demonstrated the independent association of CTRP9 with HOMA-IR (β [SE] = 97.67 [44.1]; p = 0.035). In CAD+T2DM individuals, CTRP9 was positively correlated with insulin, HOMA-IR, TNF-α, IL-6, sE-Selectin, sVCAM-1 and sICAM-1 (p < 0.01) and negatively correlated with adiponectin (p < 0.05); however, a correlation between CTRP9 and IL-6 disappeared after further adjustment for adiponectin.

**Table 3:** Pearson correlation of CTRP9 with anthropometric and metabolic parameters.

	Control (n = 80)	CAD (n = 157)	T2DM (n = 37)	T2DM+CAD (n = 63)
Age	.108	-.070	-.194	-.134
BMI	.331*	.141	.345*	.210
SBP	.169	.005	-.124	-.004
DBP	.062	-.035	-.027	.020
FBG	.242*	.039	.420**	.235
Insulin^a^	.353**	-.112	.253*	.380**
HOMA-IR^a^	.378**	-.103	.336*	.420**
TG	.134	-.011	.057	.184
TC	-.050	.018	.250	-.063
LDL-C	-.119	.001	.297	-.029
HDL-C	-.167	.125	.124	-.002
Creatinine	-.137	.048	.098	-.156
AST	.058	-.094	-.036	.124
ALT	.134	-.137	.057	.129
IL-6	.076	.273**	-.024	.325**
TNF-α^a^	-.041	.250**	.153	.365**
E-Selectin	.088	.382**	.420**	.339**
ICAM-1	.206	.403**	.362*	.421**
VCAM-1	.044	.392**	.388*	.381**
Adiponectin	-.068	-.347**	-.266*	-.262*

* Correlation is significant at the 0.05 level (2-tailed).

** Correlation is significant at the 0.01 level (2-tailed).

^a^ Logarithmic transformation was performed

Independent associations of CTRP9 with sICAM-1 (β [SE] = 0.196 [0.08]; p = 0.012), HOMA-IR (β [SE] = 74.64 [24.7]; p = 0.004) and TNF-α (β [SE] = 2.05[0.8]; p = 0.010) were shown by multiple linear regression analysis. Strikingly, in CAD patients (CAD and CAD+T2DM groups), CTRP9 was found to be significantly associated with BMI (β [SE] = 1.64 [0.815], p = 0.045), adiponectin (β [SE] = -4.37 [1.13]; p < 0.001), sE-Selectin (β [SE] = 0.757 [0.216]; p = 0.001), sICAM-1 (β [SE] = 0.142 [0.05); p = 0.003), sVCAM-1 (β [SE] = 0.091 [0.03]; p = 0.001) and TNF-α (β [SE] = 1.192 [0.53]; p = 0.026). Finally, results of multiple linear regression analysis in patients with T2DM (T2DM and CAD+T2DM groups) revealed the independent association of CTRP9 with BMI (β [SE] = 2.76 [1.17]; p = 0.021), HOMA-IR(β [SE] = 61.71 [20.54]; p = 0.003), sE-Selectin (β [SE] = 0.737 [0.34]; p = 0.033) and sVCAM-1(β [SE] = 0.084 [0.04]; p = 0.041).

## Discussion

The findings of the present study revealed that circulating CTRP9 levels were associated with an increased risk of T2DM and CAD. The associations between CTRP family members and cardio-metabolic abnormalities have been documented by previous studies [24–26]. Interestingly, we found an independent association of CTRP9 levels with soluble adhesion molecules in patients with CAD and T2DM. These results indicate that serum levels of CTRP9 were elevated in CAD and T2DM patients. Preceding studies showed increased levels of serum CTRP9 in obesity and its attendant health risks [8,18–20], while decreased serum levels of CTRP9 was reported in CAD patients [16]. These observed discrepancies may have resulted from differences such as non-CAD subject selection/exclusion criteria and ethnicities between studies.

In the current study, non-CAD subjects were selected from individuals who underwent angiography and had normal coronary arteries. Moreover, a PPAR-agonist could affect the expression of CTRP9 [27] and, therefore, patients treated with this drug (agonist) were excluded from the study. The results of this study were in line with those of Chang Hee Jung et al., who reported a positive association of CTRP9 levels with BMI and arterial stiffness [19] and a study by Asada et al. in which a positive association between serum CTRP9 levels and atherosclerosis in T2DM patients was demonstrated [18]. In contrast, decreased and increased levels of circulating CTRP9 after bariatric surgery and in patients with impaired fasting glucose were shown, respectively [20], as well as increased serum CTRP9 levels in newly diagnosed T2DM patients [28].

It has been proposed that elevated circulating CTRP9 levels in T2DM and CAD patients might be a compensatory response to insulin resistance and atherogenic milieu [19]. Notably, the current study found higher levels of CTRP9 in women than men, which is in agreement with the results of previous studies reporting a sexually dimorphic pattern of circulating CTRP9 levels [8,19]. In addition, discrepancies in the concentration range of CTRP9 levels among previous studies [19–21,29,30] may be related to the performance of various ELISA kits and the basis of their design.

CTRP9 is predominantly secreted by adipose tissue and the CTRP9 gene is up-regulated in the adipose tissue of obese mice [8]. Serum CTRP9 levels were found to be inversely correlated with BMI in individuals with T2DM [19] and reduced CTRP9 levels were observed in obese patients following bariatric surgery [20]. In the current study, circulating CTRP9 levels were independently correlated to BMI. These findings suggest that CTRP9 is up-regulated in the adipose tissue of normal and T2DM subjects compared to CAD patients, because no correlation was found between CTRP9 and BMI in CAD individuals. The favorable effects of CTRP9 on glucose metabolism and insulin sensitivity have been previously demonstrated [9,10]. Targeted deletion of CTRP9 has been shown to decrease insulin sensitivity in mice [10] and a positive association of serum CTRP9 levels with glucose metabolism parameters has been shown in human studies [19,20].

It has been suggested that CTRP9 activates Akt, AMPK and p42/44 MAPK and increases glucose uptake [31]. The current study found that CTRP9 levels are positively correlated with glucose metabolism parameters in healthy subjects and patients with T2DM (T2DM and T2DM+CAD groups), suggesting a compensatory increase in CTRP9 in insulin-resistant conditions; however, more studies are needed to prove this concept. Positive correlations between CTRP9 and inflammatory cytokines (IL-6 and TNF-α) in CAD individuals (CAD and CAD+T2DM groups) were observed in the results, indicating a compensatory response to inflammatory milieu in patients with CAD. CTRP9 inhibits inflammatory responses in macrophages and was shown to improve plaque stability by reducing inflammatory cytokine secretions in mice [31,32].

CTRP9 plays a protective role in endothelium functions as well as vasorelaxation, modulation of vascular smooth muscle cell proliferation, regulation of arterial stiffness and neointimal hyperplasia for both in vitro and in vivo models [12,14,15,19]. Moreover, CTRP9 stimulates AMP-activated protein kinase, which could inhibit expression of adhesion molecules (ICAM-1 and VCAM-1) in endothelial cells [33]. Notably, the current results revealed a positive association between CTRP9 and soluble adhesion molecules in CAD and T2DM patients. To the best of our knowledge, this is the first *in vivo* evidence for association of cell adhesion molecules with CTRP9.

It was also observed that serum levels of CTRP9 were negatively related to adiponectin in CAD patients (CAD and T2DM+CAD group). Adiponectin has anti-atherogenic properties and forms a heterodimer with CTRP9 [8]. The observed correlations between CTRP9 and outcomes of interest were significant, even after adjustment for adiponectin level, with the exception of BMI, insulin and sICAM-1 in the T2DM group and IL-6 in the CAD+T2DM group. These findings suggest that increased levels of CTRP9 may be a compensatory response to decreased adiponectin levels, insulin resistance and inflammatory milieu in CAD and T2DM patients.

In conclusion, the present study showed elevated levels of CTRP9 in T2DM and CAD patients as well as positive correlations of CTRP9 with BMI, glucose metabolism parameters, inflammatory markers and adhesion molecules and a negative correlation with adiponectin. The results of this study suggest that pathological conditions such as insulin resistance in T2DM are accompanied by a lack of sensitivity to CTRP9; yet this hypothesis should be investigated with future longitudinal, cellular and molecular biology studies.

The strength of this study lies in the selection of CAD and non-CAD patients using coronary angiographic findings as the gold standard and investigating the relation between adhesion molecules, indicators of inflammation and serum CTRP9 levels. Some limitations also should be addressed. These include the relatively small sample size, especially in the T2DM group, and a case-control study design in which the cause-and-effect relationship could not be determined.

## Supporting information

S1 Table Details of medications use. (DOCX)

S2 Table Controlling effect of covariates on CTRP9 serum levels. (DOCX)
