# Supplementary material for: Association of circulating CTRP9 with soluble adhesion molecules and inflammatory markers in patients with type 2 diabetes mellitus and coronary artery disease
Source: PLoS One. 2018 Jan 30;13(1):e0192159. doi: 10.1371/journal.pone.0192159 (PMC5790264; doi:10.1371/journal.pone.0192159)
Supplement: S1 Table — (DOCX) [file pone.0192159.s001.docx]

S-Table-1: Details of medications use in study population.

| Medications | | Control(n=80) | CAD (n=157) | T2DM (n=37) | CAD+T2DM (n=63) |
| --- | --- | --- | --- | --- | --- |
| Antihypertensive medication [n (%)] | | 11 (13.8) | 56 (35.7) | 5 (13.5) | 33 (52.4) |
|  | Angiotensin-converting enzyme inhibitors | 7 (8.75) | 50 (31.8) | 4 (10.8) | 29 (46.0) |
|  | Calcium antagonists | 2 (2.5) | 7 (4.5) | 2 (5.4) | 5 (7.9) |
|  | Angiotensin II receptor antagonists | 4 (5) | 15 (9.6) | 1 (2.7) | 12( 19.0) |
| Statin use [n (%)] | | 23 (28.7) | 88 (56.1) | 13 (35.1) | 34 (54) |
|  | Atorvastatin | 18 (22.5) | 67 (42.7) | 9 (24.3) | 29 (46.0) |
|  | Simvastatin | 5 (6.3) | 21 (13.4) | 4 (10.8) | 7 (11.1) |
| Oral hypoglycemic agent [n (%)] | | 0 | 0 | 20 (54.1) | 42 (66.7) |
|  | Metformin | 0 | 0 | 20 (54.1) | 40 (63.5) |
|  | Sulphonylureas | 0 | 0 | 7 (18.91) | 14 (22.22) |
