# Supplementary material for: Association of circulating CTRP9 with soluble adhesion molecules and inflammatory markers in patients with type 2 diabetes mellitus and coronary artery disease
Source: PLoS One. 2018 Jan 30;13(1):e0192159. doi: 10.1371/journal.pone.0192159 (PMC5790264; doi:10.1371/journal.pone.0192159)
Supplement: S2 Table — (DOCX) [file pone.0192159.s002.docx]

S-Table-2. Controlling effect of covariates on CTRP9 serum levels.

| Dependent Variable: CTRP9 | | | | | | |
| --- | --- | --- | --- | --- | --- | --- |
| (I) Group | (J) Group | Mean Difference (I-J) | Std. Error | Sig.^b^ | 95% Confidence Interval for Difference^b^ | |
|  |  |  |  |  | Lower Bound | Upper Bound |
| Control | CAD | -39.257^*^ | 7.765 | .000 | -59.868 | -18.645 |
|  | T2DM | -32.477^*^ | 10.923 | .019 | -61.472 | -3.483 |
|  | T2DM-CAD | -53.472^*^ | 9.688 | .000 | -79.187 | -27.757 |
| CAD | Control | 39.257^*^ | 7.765 | .000 | 18.645 | 59.868 |
|  | T2DM | 6.779 | 10.269 | 1.000 | -20.480 | 34.038 |
|  | T2DM-CAD | -14.215 | 8.320 | .531 | -36.302 | 7.871 |
| T2DM | Control | 32.477^*^ | 10.923 | .019 | 3.483 | 61.472 |
|  | CAD | -6.779 | 10.269 | 1.000 | -34.038 | 20.480 |
|  | T2DM-CAD | -20.994 | 10.980 | .340 | -50.140 | 8.151 |
| T2DM-CAD | Control | 53.472^*^ | 9.688 | .000 | 27.757 | 79.187 |
|  | CAD | 14.215 | 8.320 | .531 | -7.871 | 36.302 |
|  | T2DM | 20.994 | 10.980 | .340 | -8.151 | 50.140 |
| Based on estimated marginal means | | | | | | |
| *. The mean difference is significant at the .05 level. | | | | | | |
| b. Adjustment for multiple comparisons: Bonferroni. | | | | | | |
